# Loss of TRPV2 Homeostatic Control of Cell Proliferation Drives Tumor Progression

**DOI:** 10.3390/cells3010112

**Published:** 2014-02-19

**Authors:** Sonia Liberati, Maria Beatrice Morelli, Consuelo Amantini, Valerio Farfariello, Matteo Santoni, Alessandro Conti, Massimo Nabissi, Stefano Cascinu, Giorgio Santoni

**Affiliations:** 1 School of Pharmacy, Section of Experimental Medicine, University of Camerino, P.zza dei Costanti, 63032, Camerino, Macerata, Italy; E-Mails: sonia.liberati@uniroma1.it (S.L.); beatricemorelli@hotmail.com (M.B.M.); consuelo.amantini@unicam.it (C.A.); valerio.farfariello@uniroma1.it (V.F.); massimo.nabissi@unicam.it (M.N.); giorgio.santoni@unicam.it (G.S.); 2 Department of Molecular Medicine, Sapienza University, Viale Regina Elena 291, 00161, Rome, Italy; 3 Medical Oncology, Polytechnic University of the Marche Region, Via Tronto 10, 60020, Ancona, Italy; E-Mails: alessandro.conti@hotmail.com (A.C.); cascinu@yahoo.com (S.C.); 4 Department of Medical Oncology, “Ospedali Riuniti” University Hospital, Polytechnic University of the Marche, Via Tronto 10/a, Torrette, Ancona, Italy

**Keywords:** Transient Receptor Potential Channels, Transient Receptor Potential Vanilloid-type 2, tumor progression, glioblastoma, prostate cancer, transitional cell carcinoma of human bladder, hepatocarcinoma

## Abstract

Herein we evaluate the involvement of the TRPV2 channel, belonging to the Transient Receptor Potential Vanilloid channel family (TRPVs), in development and progression of different tumor types. In normal cells, the activation of TRPV2 channels by growth factors, hormones, and endocannabinoids induces a translocation of the receptor from the endosomal compartment to the plasma membrane, which results in abrogation of cell proliferation and induction of cell death. Consequently, loss or inactivation of TRPV2 signaling (e.g., glioblastomas), induces unchecked proliferation, resistance to apoptotic signals and increased resistance to CD95-induced apoptotic cell death. On the other hand, in prostate cancer cells, Ca^2+^-dependent activation of TRPV2 induced by lysophospholipids increases the invasion of tumor cells. In addition, the progression of prostate cancer to the castration-resistant phenotype is characterized by *de novo* TRPV2 expression, with higher TRPV2 transcript levels in patients with metastatic cancer. Finally, TRPV2 functional expression in tumor cells can also depend on the presence of alternative splice variants of TRPV2 mRNA that act as dominant-negative mutant of wild-type TRPV2 channels, by inhibiting its trafficking and translocation to the plasma membrane. In conclusion, as TRP channels are altered in human cancers, and their blockage impair tumor progression, they appear to be a very promising targets for early diagnosis and chemotherapy.

## 1. Introduction

Transient receptor potential (TRP) cation channels are unique cellular sensors characterized by a promiscuous activation mechanism [1]. More than 50 members of the TRP family have been characterized in yeast, worms, insects, fish, and mammals [2], making them one of the largest groups of ion channels. The 33 mammalian TRPs identified thus far can be sorted into seven subfamilies: TRPC (Canonical), TRPM (Melastatin), TRPV (Vanilloid), TRPA (Ankyrin transmembrane protein), TRPP (Polycystin), TRPML (Mucolipin) and TRPN (NomPC-like). TRPs are classified essentially according to their primary amino acid sequence rather than selectivity or ligand affinity, as their properties are heterogenous and their regulation is complex. From a structural standpoint, TRP channels are membrane proteins with six putative transmembrane spans (TMs) and a cation-permeable pore region formed by a short hydrophobic stretch between TM5 and TM6. The structural differences within the TRP subfamilies have been recently reviewed [3]. The TRP proteins are essentially cation-permeable ion channels sensitive to a remarkable range of stimuli. 

Neoplastic transformation is the result of the accumulation of mutations in certain key signaling proteins, along with formation and selection of more aggressive cancer sub-clones. Researchers have found that among these proteins, the ion channels belonging to the TRP super-family profoundly affect a variety of pathological processes [1,4]. Studies over the last 10 years have progressively clarified the extent to which TRP channels are associated with cancer, and have shed light on the involvement of these receptors in triggering enhanced proliferation, aberrant differentiation, and impaired ability to die, which lead to uncontrolled cancer expansion and tumor invasion [5]. Moreover, further understanding of changes in TRP channel expression in cancer cells will be obtained as we gain knowledge about the molecular events involved in cancer progression of TRP channels.

The expression levels and activity of members of the TRPC, TRPM, and TRPV families have been correlated with tumor growth and progression [6]. Malignant cell transformation is often accompanied by changes in ion channel expression. Depending on the stage of the cancer, either increased or decreased expression of TRP mRNA and protein levels have been reported. These changes may have cancer promoting effects by increasing the expression of constitutively active TRP channels in the plasma membrane of cancer cells, thus enhancing Ca^2+^-dependent proliferative response or may offer a survival advantage, such as resistance of cancer cells to apoptotic-induced cell death [6]. In addition, a role for TRP gene mutations in cancer development, and a relationship between the expression of specific TRP gene single nucleotide polymorphisms (SNPs) and increased cancer risk have been recently reported [7].

Finally, over the past decade, an increasing set of data has revealed that changes in TRP channel mRNA and protein expression might represent valuable diagnostic and/or prognostic markers, as well as targets for pharmaceutical intervention and targeting in cancer [5,8].

## 2. Structure, Expression and Functional Activation of TRPV2 Channels.

The TRPV subfamily (vanilloid receptors) comprises channels critically involved in nociception and thermosensing (TRPV1, TRPV2, TRPV3, and TRPV4), and renal Ca^2+^ absorption/reabsorption (TRPV5 and TRPV6). Apart from TRPV1, the pharmacology of these channels is still insufficiently known; furthermore, only a few small-molecule ligands have been identified, and little is known about their endogenous ligands, resulting in a substantial “orphan” state for these channels [9]. 

Among these members of the TRPV channel family, TRPV2 is a non-selective cation channel showing Ca^2+^ permeability. Structurally, it is composed of six transmembrane domains, a putative pore-loop region, a cytoplasmic amino terminus with three ankyrin-repeat domains, and a cytoplasmic carboxy terminus [10,11]. TRPV2 functions as a tetramer, and each monomer contains 761 residues, with a large N-terminal region of 389 amino acids, a smaller 250-residue TM domain, and a 122-residue C-terminal region. The 3D structure of TRPV2 reveals a “hanging gondola architecture” [12]. Functional studies have evidenced that TRPV2 is activated by noxious heat, with an activation threshold greater than 52 °C, and by a number of exogenous chemical ligands, showing species specificity. Very recently, several novel cannabinoid TRPV2 modulators were identified. Thus, Cannabidiol (CBD), a nonpsychotropic compound, the psychotropic agent Δ^9^-tetrahydrocannabinol (Δ^9^-THC) found in *Cannabis sativa* L. and cannabinol (CBN), were found to be potent TRPV2 agonists [13,14,15]. TRPV2 has been proposed as a potential pain target, but very little is known about its activation mechanism or possible candidates as specific or endogenous activators. TRPV2 was found to be expressed, both in the plasma membrane and in the early endosome [16,17]. Many studies have suggested that activation of TRPV2 by growth factors causes phosphatidylinositol 3 kinase (PI3K)-dependent and independent translocation of the channel to the plasma membrane [18,19]. In non-neuronal cell lines, such as pancreatic MIN6 or CHO cells [20], growth factors present in serum (e.g., insulin-like growth factor-1, (IGF-1)) up-regulate TRPV2 expression and function by inducing a dynamic and transient translocation of the TRPV2 channel from intracellular compartments to the plasma membrane through a PI3K-dependent pathway [18,21]. In accordance with this scenario, the PI3K inhibitors LY2934001 could block the translocation of TRPV2 to the plasma membrane. In addition, IGF-1, heat, platelet-derived growth factor (PDGF), and CBD promote TRPV2 membrane insertion. Finally, the recent use of anti-TRPV2 mAb has dispelled the controversy on the contribute of IGF-1, on TRPV2 expression, by demonstrating that this growth factor has no effect in the trafficking of this channel to the plasma membrane [22]. 

In addition, HEK-293 cells transfected with plasmids encoding mouse TRPV2 (mTRPV2) channel [19] displayed a spread out morphology with vacuolated cytoplasm and numerous filopodia. These morphological characteristics of mTRPV2-expressing cells are typical of cellular death and suggested that expression of TRPV2 could have cytotoxic effects. These events could be prevented by reducing extracellular calcium concentration or when a mutant mTRPV2 channel, carrying a charge substitution (Glu^594^ to Lys^594^) in the pore-forming domain, was expressed in HEK-293 cells, indicating that cellular toxicity was directly linked to mTRPV2 channel activity. TRPV2-mediated cytotoxicity could also be prevented by inhibition of PI3K with chemical inhibitors or by serum deprivation. In stably transfected CHO cells, it was found that mTRPV2, but not the pore mutant channels, displayed a constitutive activity that resulted in increased resting calcium levels, events that could also be prevented by serum starvation.

## 3. Role of TRPV2 Expression in Different Cell Types of Tumor

There is increasing evidence that several members of the TRP ion channels may play opposing role (oncogenic *versus* tumor suppressor) during carcinogenesis. In addition, to further complicate the matter, the cancerogenic effects of TRP channels are cell type-dependent, that is a TRP channel, which is oncogenic in one cell type, may be conversely a tumor suppressor in another cell. Moreover, changes on TRP channel expression and function may be also the result of the presence of specific TRP gene single nucleotide polymorphisms (SNPs), mRNA splice variants, different activation state and subcellular localization of TRP channels, *etc.* Thus, due to the complexity of the TRP channel family in tumors, to facilitate the reader, a table relative to the oncogenic and tumor suppressor effects of TRPV2 in different cancer types has been presented at the end of this review (Table 1).

**Table 1:** Oncogenic and Tumor suppressor effects of TRPV2 expression in different tumors.

Tumor	Oncogenic	Tumor suppressor	Reference
Mantle Cell Lymphoma	+		[23]
Multiple Myeloma	+		[24]
Myeloid Acute Leukemia		−	[25]
Glioblastoma		−	[26]
Bladder Carcinoma	+		[27]
Prostate Adenocarcinoma	+		[28]
Hepatocarcinoma	+		[29]

(+) up-regulation of TRPV2 expression; (−) down-regulation of TRPV2 expression.

### 3.1. TRPV2 in Lymphomas, Leukemias, and Multiple Myelomas

The analysis of human transcrittoma by GNF gene expression has indicated the expression of TRPV2 channels in CD34^+^/CD45^+^/CD133^+^CD73^−^ haematopoietic stem cells [30], suggesting a role for these channels in haematopoietic-derived leukaemia and lymphoma tumors.

The region 17p11.2, where the human TRPV2 gene maps, is an unstable chromosomal region characterized by a large number of low-copy repeats, which have been proven to mediate deletion and duplication in several genomic disorders and amplifications in hematological cancers [31]. Thus, loss or gain of the TRPV2 gene has been associated with cancer growth and progression. A role of TRPV2 gene mutations in hematopoietic cancer development has been recently reported. Aberrant TRPV2 expression in B lymphocytes from mantle cell lymphoma (MCL) cell lines and tissues has been reported [23]. MCL is a B cells non-Hodgkin lymphoma with a poor prognosis and a median survival time of about three to five years [23] characterized by malignant transformation of the mantle zone cells surrounding the germinal center. Among the transmembrane proteins expressed in the MCL plasma membrane, TRPV2 and hydrogen voltage-gated channel 1 (HVCN1) ion channels were found. The HVCN1 co-localizes with the B cell receptor and is involved in class switch recombination [32]; TRPV2 was identified in the plasma membrane of Z138 MCL-derived cell lines [33] but its role is still unknown. 

TRPV2 overexpression was also evidenced by microarray analysis in multiple myeloma (MM) patients [34]. MM is a malignancy of clonal bone marrow plasma cells characterized by a high genomic instability that increases with disease progression. The 17p11.2-p12 amplified region was detected in the KMS-26 myeloma cell line by a SNP microarray. Analysis of transcriptional profiles in a proprietary database of myeloma cell lines identified 12 significantly overexpressed genes in the KMS-26 amplified region, including TNFRSF13B/TACI, COPS3, NCOR1 and TRPV2, and the TNFRSF13B/TACI gene showed the highest average fold-change (>8); it encodes a receptor for the B-cell activating factor (BAFF) and the proliferation-inducing ligand (APRIL), which are thought to contribute to survival of MM cells.

In this regard, a recent study conducted in our laboratory has reported the presence of two distinct myeloma cell subpopulations in MM patients showing different TRPV2 phenotypes: the CD138^+ ^TRPV2^+^ and CD138^+^ TRPV2^−^ MM cells. In addition, the TRPV2 agonist, cannabidiol (CBD), a non-psychoactive cannabinoid with anti-tumoral activity, alone, or in combination with bortezomib, strongly inhibited cell growth, arrested cell cycle progression, and induced cell death of MM cells, by regulating the ERK, AKT and NF-κB signaling pathways, with the major effects observed in the RPMI8226 and U266 MM cell lines transfected with TRPV2, compared to the untransfected RPMI8226 and U266 cell lines [24].

In B malignancy TRPV2 mRNA expression was also found in Burkitt Lymphoma (Raji and Daudi cell lines) [35], Epstein-Barr virus-transformed B-cell line, LCL 721, lacks HLA-A, -B, and -C class I antigens and transcripts [36]. In addition, TRPV2 down-regulation has been described in acute myeloid leukemia and myelodisplastic syndrome (AML/MDS) patients [25].

### 3.2. TRPV2 Expression in Gliomas

Diffusely infiltrating gliomas are the most common primary brain tumors. They are characterized by diffuse infiltration of adjacent brain structure and tendency for progression to anaplasia over the time. Glioblastoma Multiforme GBM represents the end of the spectrum of malignancy of these neoplasms [37,38]. Despite advances in medical and surgical therapies, the outcome for patients diagnosed with GBM remains dismal. Two major features of GBM aggressiveness are high proliferation rate and resistance to chemotherapeutic drug-induced apoptosis [39]. In this regard, we have provided evidence of the expression of TRPV2 by glioma cells and tissues and its involvement in ERK-dependent regulation of glioma cell proliferation and susceptibility to Fas-induced apoptosis [26]. TRPV2 was found to be expressed in benign astrocyte tissues, in glioma cell lines and in glioma tissues, with a marked reduction of TRPV2 in high-grade gliomas, suggesting that TRPV2 expression played a negative role in tumor progression. Moreover, silencing of the TRPV2 gene in U87MG cell line resulted in the modulation of genes promoting cell proliferation and survival, accompanied by a marked increase in cell survival and proliferation. The increased proliferation was mainly sustained by cells expressing the neuronal β_III_-tubulin marker compared to GFAP^+^ cells of astrocytic origin. In addition, enforced TRPV2 expression on low TRPV2-expressing MZC glioma cells resulted in a marked inhibition of cell survival and enhanced spontaneous apoptosis. 

Glioma cells are moderately sensitive to Fas-induced cell death [40], and this sensitivity might be increased by treatment with some antitumor chemotherapeutic agents [41]. We also found that, in accordance with down-modulation of Fas expression and up-regulation of Bcl-X_L_, TRPV2 silencing in U87MG cells increased their resistance to apoptotic cell death triggered by anti-Fas-specific mAb used alone or in combination with suboptimal doses of anticancer agents, such as VP-16 and BCNU [41]. ERK activation is a key event controlling survival and proliferation of different cancer cells including glioma [42]. In this regard, we have demonstrated that TRPV2 silencing of U87MG cells is associated with increased ERK phosphorylation. Inhibition of ERK activation by PD98059 treatment markedly reduced cell viability and proliferation in siTRPV2-U87MG transfected cells. PD98059 significantly increased Fas expression and triggering in siTRPV2-U87MG transfected cells. Accordingly, inhibition of ERK activation was associated with a reduction of Bcl-X_L_ levels and with an increased sensitivity of siTRPV2 glioma cells to Fas-induced mitochondrial-dependent apoptosis, with the maximal effects observed by using anti-Fas mAb in combination with VP-16 or BCNU [26]. 

A recent study demonstrated that the TRPV2 agonist CBD was able to enhance TRPV2 expression and activation leading to an increased chemosensitivity of glioma cells [43]. We demonstrated that CBD, by inducing TRPV2 channel activation, was able to increased drug permeation. By using natural red fluorescent Doxorubicin (DOXO), we showed that its uptake is markedly increased in TRPV2 overexpressing MZC glioma cells. Moreover the treatment of these cells with CBD strongly promoted drug uptake. In addition, the CBD mediated effects were completely inhibited by EGTA, indicating an important role for Ca^2+^. These results suggested that CBD might cause a conformational change in the pore helix structure favouring extracellular DOXO uptake. Therefore we created a construct expressing a TRPV2 protein lacking the complete pore domain region (572–609 aa). Glioma cells expressing this mutant TRPV2 channel showed an inhibition of TRPV2-induced cytotoxic effects and reduced DOXO uptake. Since the chemotherapeutic drugs used in this study have a molecular weight lower than DOXO (DOXO MW: 580; TMZ MW: 194; BCNU MW: 214), it suggested that TRPV2 channels can also mediate their uptake in glioma cells. In conclusion these data give strong biological evidences that could open the development of new effective approaches based on the TRPV2 channel for the treatment of GBM. 

**Figure 1:**
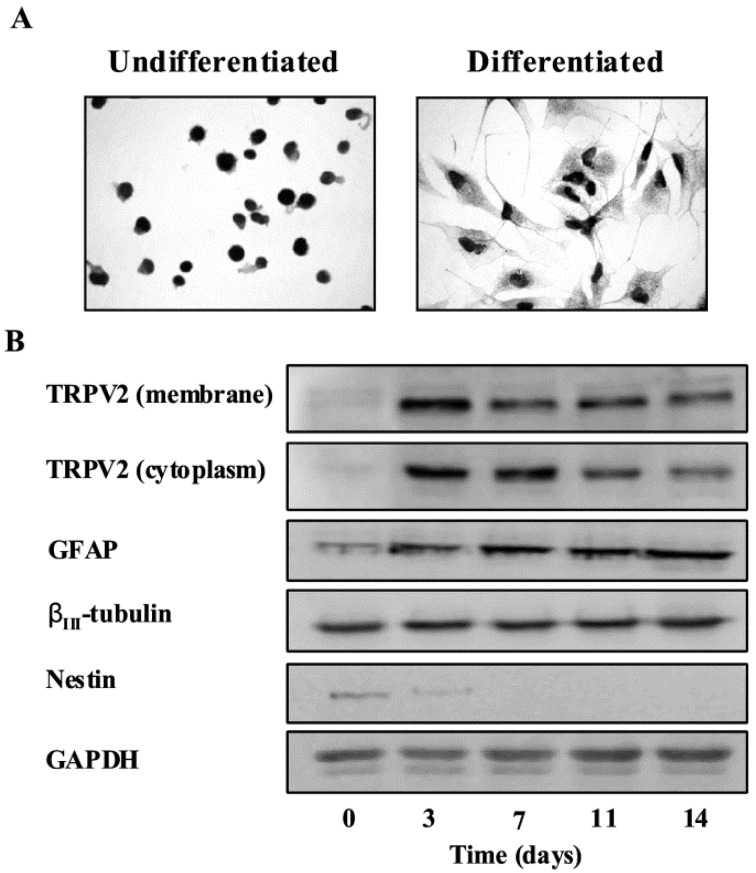
TRPV2 protein expression during GSC differentiation. (**A**) TRPV2 protein expression in undifferentiated and differentiated GSCs was evaluated by immunocytochemistry analysis. GSCs were incubated with goat anti-TRPV2 antibody followed by the respective secondary antibody. The detection was performed by avidin-biotin complex peroxidase method and diaminobenzidine (DAB) as a chromogen. The result shown that a moderate reaction that stains all the cytoplasm occurs in differentiated GSCs compared to the undifferentiated GSCs. (**B**) Membrane and cytoplasm fractions from undifferentiated or differentiated GSCs were immunoblotted with anti-TRPV2 antibody. Total lysates were also immunoblotted with anti-GFAP, anti-βIII-tubulin and anti-nestin antibodies. GAPDH protein was used as loading control.

Adapted from 1 [54] Copyright John Wiley and Sons 2012. Permission obtained from copyright holder.

While neoplastic transformation of differentiated glial cells was for many years the most accepted hypothesis to explain the origin of gliomas [44,45], recent findings support the existence of a stem cell-derived origin for different types of cancers including brain tumors [45,46]. In particular, glioblastoma stem-like cells (GSCs) have been isolated from both human tumor tissues [47,48,49,50,51] and several glioma cell lines [49,52,53]. We have demonstrated that TRPV2 is expressed in GSC lines and that their differentiation was associated with an increase of TRPV2 levels [54]. GSC differentiation induces a marked enhancement of TRPV2 protein, both in the cytosol and in the plasma membrane compartments (Figure 1).

The TRP antagonist, ruthenium red (RR), markedly impaired GSC differentiation as evaluated by the reduced expression of GFAP and β_III_-tubulin, markers of differentiation. Also the knockdown of TRPV2 gene reduced the percentage of GFAP^+^ and β_III_-tubulin GSC subpopulations. By contrast, TRPV2 overexpression in undifferentiated GSC-transfected cell lines resulted in a more mature glial phenotype associated with a reduced proliferation. Tumor xenografts derived from TRPV2-overexpressing GSC lines showed a significant reduction in diameter and mitotic index, associated with a GFAP-positive differentiated morphology. Interestingly, TRPV2-expressing tumors evidenced the presence of several GFAP mononuclear-giant cells (MNGCs) resembling those found in GBM subtypes showing low proliferative rate and better prognosis [54,55,56,57]. Therefore, these results suggest for a role of TRPV2 in GSC-derived tumor progression.

### 3.3. TRPV2 Expression in Bladder Tumor Cells

TRPV2 expression and activity could depend on the presence of alternative splicing mRNA variants, resulting in multiple channel proteins. Thus, reduction of loss of these splice variants during tumor progression tracking is associated with the acquisition of an invasive tumor phenotype and malignant behavior [27]. TRPV2 expression and activity in urothelial bladder cancer cells (UC) can depend on the presence of alternative splicing variants, resulting in multiple channel proteins. As evaluated by PCR, two TRPV2 transcripts, the hTRPV2, and the s-TRPV2 variants, are expressed in both normal human urothelial cells (NHUCs) and normal bladder tissues. Sequencing analysis has demonstrated that s-TRPV2 represents a novel alternative splicing variant of TRPV2. Cytofluorimetric and confocal analyses have revealed that TRPV2 protein was expressed both in cytoplasm and plasma membrane of NHUCs and was identified as a doublet of 97 and 82 kD [27], likely corresponding to the monomeric and short form of TRPV2, respectively.

**Figure 2:**
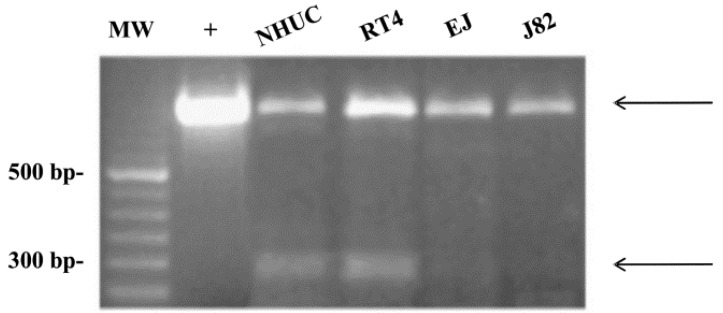
s-TRPV2 expression in human normal urothelial and urothelial carcinoma cell lines. cDNA from NHUCs and RT4, EJ, and J82 cell lines was amplified by PCR for TRPV2 expression. PCR products were analyzed by electrophoresis on 2% ethidium bromide-stained agarose gel. The hTRPV2-plasmid construct was used as positive control (+). Arrows indicate the two products obtained. MW = molecular weight.

Adapted from [27] Copyright Elsevier, 2008. Permission obtained from copyright holder.

By immunohistochemistry, we also showed that in normal bladder tissues, TRPV2 localized in the superficial cells, whereas, negligible or no expression was observed in basal and club-shaped cells. In addition, we found that TRPV2 protein is expressed in both low-grade RT4 and high-grade EJ and J82 cell lines, with higher expression observed in J82 cells. Notably, confocal microscopy showed that TRPV2 staining was granular and localized in the cytoplasm and plasma membrane of UCs. The analysis of TRPV2 gene expression in superficial and invasive UCs with distinct grades and stages indicates that TRPV2 mRNA and protein expression progressively increases as grade and stage increase, with stronger expression in G3 pT4 UC specimens, suggesting that receptor expression can be modulated during urothelial cell differentiation. Moreover, the s-TRPV2 variant was expressed in low-grade RT4 cells, and irrespective of grade of malignancy its expression progressively decreased in pTa, pT1, and pT2 and was undetectable in high grade EJ and J82 cells, and pT3 and pT4 invasive bladder cancer tissues (Figure 2).

As demonstrated for TRPV1 channel, the s-TRPV2 splice variants [27] may act as dominant-negative mutants by forming heterodimers with wild-type TRPV2 and inhibiting its trafficking and translocation to the plasma membrane [58]. Finally, we also observed significant differences in the short/full TRPV2 form ratio from NB to pTa and pT1 stages, strongly suggesting that loss of s-TRPV2 may represent an early event in bladder carcinogenesis, and that the enhanced TRPV2 expression in high-staged muscle-invasive UC (pT2-T4) could be secondary to this event. Similar to our study, a different s-TRPV2 lacking the pore-forming region and sixth transmembrane domain has been recently identified in human leukemic cells [59]. Finally, TRPV2 mRNA has been found to be abundantly expressed in T24 UC cells, and the expression level in UC cells was correlated with high-grade disease. The administration of the TRPV2 agonist, CBD, increased intracellular calcium concentrations in T24 UC cells. CBD progressively decreased the viability of T24 cells in a dose-dependent manner, whereas RT4 UC cells were mostly unaffected. Cell death induced by CBD occurred via apoptosis caused by continuous influx of calcium through TRPV2 [60], suggesting that TRPV2 channels in UC cells may represent a potential new therapeutic target, especially in higher-grade UC cells. Furthermore, a recent study using 5637 bladder cancer cells transfected with rat TRPV2 cDNA, showed that TRPV2 overexpression enhanced bladder cancer cell migration and invasion by direct matrix metalloproteinase 2 (MMP2) regulation [61].

Other potential mechanisms linking TRPV2 to tumor development could be related to its ability to physically interact with proteins involved in cell proliferation [62] and sensitivity to stretch stimuli. In mouse, TRPV2 is activated by IGF-1, a cytokine endowed with potent mitogenic and anti-apoptotic effects on cancer cells [18,63,64]; moreover, increased proliferation and survival of human bladder smooth muscle cells, induced by mechanical stress, is associated with increased IGF-1 levels [65]. Given the high level of IGF-1/IGF-1 receptor (IGF-1R) found in bladder tumors and its role in bladder cancer progression [66], and the strong TRPV2 increase in high-grade and -stage tumors [27], we suggest that the TRPV2/ IGF-1/IGF-1R pathway plays an important role in the control of UC growth and progression.

The pathophysiologic relevance of TRPV2 expression both in normal urothelium and UC of the human bladder is presently unknown. The recently proposed role for TRPV2 as an endogenous sensor of noxious heat and mechanical stretch [67] is in line with the view of a sensory role of urothelium [68]. Consequently, urothelial TRPV2 may also represent a drug target for urinary bladder pain [67]. The ability of tetrahydrocannabinol to activate human TRPV2 (hTRPV2) [14] and our finding of higher TRPV2 expression in invasive bladder tumors together with the palliative use of cannabinoids in cancer treatment [69] could encourage the use of THC as an adjuvant in the treatment of severe and persistent bladder cancer pain.

### 3.4. TRPV2 Expression in Prostate Cancers

Activation of TRPV2 channels by growth factors, hormones, serum, and agonists induces a dynamic and transient translocation of TRPV2 channels from intracellular compartments (early endosome) to the plasma membrane, which results in the development of death signals [18,21]. By contrast, in some tumors, the overexpression of TRPV2 channels results in activation of aberrant signaling pathways that drives unchecked cell proliferation and resistance to apoptotic stimuli. Thus, the loss of homeostatic controls of survival and growth factor-dependent proliferation exerted by TRPV2 channels result in a proliferative advantage for cancer cells [26].

A role for TRPV2 in the invasive capability of prostate cancer cells has been recently reported. Ca^2+^-dependent activation and translocation of TRPV2 by lysophospholipids (LPL), such as lysophosphatidylcoline and lysophosphatidylinositol, resulted in increased PC3 prostate tumor cell migration, and involved Gq/Go-protein and PI3,4-K pathways. Silencing of the TRPV2 gene in PC3 cells or inhibition of the PI3,4-K pathway abolished the stimulatory effects of LPL on both calcium entry and migration [28]. TRPV2-regulated cytosolic calcium levels promoted PC3 migration by induction of key proteases, namely matrix metalloproteinase-2 (MMP-2), MMP-9 and cathepsin B. The expression profile of some TRP channels changes during the development and the progression of prostate cancer towards hormone-refractory stages [70]. The constitutive activity of TRPV2 is also critical for castration-resistant prostate cancer development and progression *in vivo*. Indeed, the progression of prostate cancer to the castration-resistant phenotype is characterized by *de novo* expression of TRPV2, and higher levels of TRPV2 transcripts are found in patients with metastatic cancer (stage M1) as compared to primary solid tumors (stage T2a and T2b) [71]. In addition, induction of MMP-9 and cathepsin B expression was observed upon transfection of TRPV2 in the LNCaP cell line, and knockout of TRPV2 by siRNA reduced the growth and the invasive properties of PC3 cells by progressive down-regulation of MMP-2, MMP-9 and cathepsin B expression in a xenograft tumor model [71]. 

### 3.5. TRPV2 Expression in Hepatocarcinoma

Hepatocellular carcinoma (HCC) is the fifth most common human cancer and third most frequent cause of cancer death worldwide [72]. Despite the discovery of several carcinogenetic pathways, the mechanisms of liver carcinogenesis still remain to be clarified [73]. Cirrhosis, caused by Hepatitis C virus and Hepatitis B virus infection, represent the most commonly known risk factor for hepatocellular carcinoma and, therefore, has been considered a pre-malignant condition [29,74,75]. A number of *in vivo* studies have examined the role of chronic inflammation and cytokines in the development of fibrosis, liver cell proliferation and malignant transformation [76]. Up-regulation of the TRPV2 protein levels were reported in dorsal root ganglion neurons after intraplantar injection of complete Freund’s adjuvant, suggesting a TRPV2 role in peripheral sensitization during inflammation or peripheral nerve injury [77,78]. It can be hypothesized that induction of TRPV2 may be attributable to chronic inflammation in chronic hepatitis and cirrhotic livers. TRPV2 overexpression at both the mRNA and protein levels was found in cirrhotic livers compared with those with chronic hepatitis. Moreover, the expression of TRPV2 is associated with human HCC progression. Up-regulation of TRPV2 at both mRNA and protein levels, which inversely correlated with histopathological differentiation, indicates a role for TRPV2 in hepatocarcinogenesis. In addition, a correlation between TRPV2 expression and portal vein invasion of HCC was found. Portal vein involvement in HCC patients usually predicts poor prognosis for the disease [79,80,81]. Angiogenesis and disruption of liver vascular architecture have been linked to the progression of cirrhosis and HCC in chronic liver diseases, by increasing hepatic vascular resistance and portal hypertension and decreasing hepatocyte perfusion [82].

## 4. Conclusion

In normal cells, TRPV2 expression exerts a negative homeostatic control of aberrant proliferation by inducing apoptotic cell death; loss or gain of TRPV2 gene and environmental-induced changes of TRPV2 expression result in unchecked proliferation, loss of cell differentiation and invasiveness. In this regard, it is plausible that TRPV2 plays a major role in the first events that parallel the transformation of a single normal stem/progenitor cell into a tumor cell; moreover, the TRPV2 expression was also modulated during the carcinogenesis process that follows a long and continuous exposure to different carcinogenetic agents, resulting in tumor growth and progression and selection of more aggressive tumor clones. The possibility to use TRPV2 in cancer therapy is still in infancy. TRPV2 has been shown to play important roles in skeletal, cardiac, muscular, immune, and neuronal physiology. Thus, a therapy targeting this receptor must be taken in consideration for the potential side-effects. However, the recent determination of the 3D (three-dimensional) TRPV2 structure and channel functions [12], the availability of specific monoclonal antibodies [31], and the recent preliminary *in vitro* findings on the possibility to stratify the susceptibility of myeloma cells to the CBD and/or bortezomib effects on the basis of TRPV2 expression, and the selectivity of a TRPV2 agonist (e.g., CBD), to specifically affect the CD138^+^TRPV2^+^ myeloma cells, with respect to the CD34^+^TRPV2^+^ hematopoietic progenitor cells used as negative control [24], open new prospective and hope for the application of the TRPV2 target therapy in cancer in the new future.

## List of Abbreviations

CBD: Cannabidiol
DOXO: Doxorubicin
GBM: glioblastoma multiforme
GSC: glioblastoma stem-like cells
HCC: hepatocellular carcinoma
MCL: mantle cell lymphoma
MMP: matrix metalloproteinase
PI-K: phosphatidylinositol kinase
TRP: Transient receptor potential
TRPV: TRP Vanilloid
UC: urothelial cancer cells
