# Correction: Liberati, S., *et al.* Loss of TRPV2 Homeostatic Control of Cell Proliferation Drives Tumor Progression. *Cells* 2014, *3*, 112–128

**DOI:** 10.3390/cells3020660

**Published:** 2014-06-20

**Authors:** Sonia Liberati, Maria Beatrice Morelli, Consuelo Amantini, Valerio Farfariello, Matteo Santoni, Alessandro Conti, Massimo Nabissi, Stefano Cascinu, Giorgio Santoni

**Affiliations:** 1 School of Pharmacy, Section of Experimental Medicine, University of Camerino, P.zza dei Costanti, 63032, Camerino, Macerata, Italy; E-Mails: sonia.liberati@uniroma1.it (S.L.); beatricemorelli@hotmail.com (M.B.M.); consuelo.amantini@unicam.it (C.A.); valerio.farfariello@uniroma1.it (V.F.); massimo.nabissi@unicam.it (M.N.); giorgio.santoni@unicam.it (G.S.); 2 Department of Molecular Medicine, Sapienza University, Viale Regina Elena 291, 00161, Rome, Italy; 3 Medical Oncology, Polytechnic University of the Marche Region, Via Tronto 10, 60020, Ancona, Italy; E-Mails: alessandro.conti@hotmail.com (A.C.); cascinu@yahoo.com (S.C.); 4 Department of Medical Oncology, “Ospedali Riuniti” University Hospital, Polytechnic University of the Marche, Via Tronto 10/a, Torrette, Ancona, Italy

The authors wish to make the following corrections to this paper [1]:

On p.113, we say that: “The 33 mammalian TRPs identified thus far can be sorted into seven subfamilies: TRPC (Canonical), TRPM (Melastatin), TRPV (Vanilloid), TRPA (Ankyrin transmembrane protein), TRPP (Polycystin), TRPML (Mucolipin) and TRPN (NomPC-like).” However, TRPN channels have not yet been detected in mammals. Therefore, as of today, mammalian TRP channels belong to six subfamilies: TRP canonical (TRPC), TRP vanilloid (TRPV), TRP melastatin (TRPM), TRP ankyrin (TRPA), TRP polycystin (TRPP), and TRP mucolipin (TRPML).

These changes have no material impact on the conclusions of our paper. 

The authors would like to apologize for any inconvenience this has caused the readers.
